# Malaria elimination in remote communities requires integration of malaria control activities into general health care: an observational study and interrupted time series analysis in Myanmar

**DOI:** 10.1186/s12916-018-1172-x

**Published:** 2018-10-22

**Authors:** Alistair R. D. McLean, Hla Phyo Wai, Aung Myat Thu, Zay Soe Khant, Chanida Indrasuta, Elizabeth A. Ashley, Thar Tun Kyaw, Nicholas P. J. Day, Arjen Dondorp, Nicholas J. White, Frank M. Smithuis

**Affiliations:** 1 Medical Action Myanmar, Yangon, Myanmar; 2 Myanmar Oxford Clinical Research Unit (MOCRU), Yangon, Myanmar; 3 Department of Public Health, Ministry of Health and Sports, Nay Pyi Taw, Myanmar; 4 Mahidol-Oxford Tropical Medicine Research Unit (MORU), Faculty of Tropical Medicine, Mahidol University, Bangkok, Thailand; 5 Centre for Tropical Medicine and Global Health, Nuffield Department of Clinical Medicine, University of Oxford, Oxford, UK

**Keywords:** *P*. *falciparum*, *P*. *vivax*, Malaria, Community health workers, Vertical integration, Health systems strengthening, Myanmar, Sustainability, Elimination

## Abstract

**Background:**

Community health workers (CHWs) can provide diagnosis and treatment of malaria in remote rural areas and are therefore key to the elimination of malaria. However, as incidence declines, uptake of their services could be compromised if they only treat malaria.

**Methods:**

We conducted a retrospective analysis of 571,286 malaria rapid diagnostic tests conducted between 2011 and 2016 by 1335 CHWs supported by Medical Action Myanmar. We assessed rates of decline in *Plasmodium falciparum* and *Plasmodium vivax* incidence and rapid diagnostic test (RDT) positivity rates using negative binomial mixed effects models. We investigated whether broadening the CHW remit to provide a basic health care (BHC) package was associated with a change in malaria blood examination rates.

**Results:**

Communities with CHWs providing malaria diagnosis and treatment experienced declines in *P*. *falciparum* and *P*. *vivax* malaria incidence of 70% (95% CI 66–73%) and 64% (59–68%) respectively each year of operation. RDT positivity rates declined similarly with declines of 70% (95% CI 66–73%) for *P*. *falciparum* and 65% (95% CI 61–69%) for *P*. *vivax* with each year of CHW operation. In four cohorts studied, adding a BHC package was associated with an immediate and sustained increase in blood examination rates (step-change rate ratios 2.3 (95% CI 2.0–2.6), 5.4 (95% CI 4.0–7.3), 1.7 (95% CI 1.4–2.1), and 1.1 (95% CI 1.0.1.3)).

**Conclusions:**

CHWs have overseen dramatic declines in *P*. *falciparum* and *P*. *vivax* malaria in rural Myanmar. Expanding their remit to general health care has sustained community uptake of malaria services. In similar settings, expanding health services offered by CHWs beyond malaria testing and treatment can improve rural health care while ensuring continued progress towards the elimination of malaria.

**Electronic supplementary material:**

The online version of this article (10.1186/s12916-018-1172-x) contains supplementary material, which is available to authorized users.

## Background

The global burden of malaria has fallen substantially since the beginning of the millennium (37% global decrease between 2000 and 2015), primarily due to improvements in access to diagnosis and treatment and increased coverage of insecticide-treated bednets [1]. Many of the estimated 216 million malaria cases and 445,000 deaths in 2016 occurred in remote areas of the tropics where health services are weak or non-existent [2]. Myanmar has the greatest burden of malaria in the Greater Mekong Subregion. Much of the burden of malaria is borne by remote and hard-to-reach communities [2]. The health care system in these communities has been weakened by decades of conflict and under-investment. The infrastructure is poor, and most villagers have no access to trained health staff and resort to informal health care providers (known by the colloquial term “quack” in Myanmar). These providers are generally untrained villagers who sell medicines unofficially. Over the past 6 years, investment in rural health care services has increased substantially [3], supported by both the national government and international donors, in particular from *The 3 Millennium Development Goal Fund* (a consortium of bilateral donors for Myanmar) and *The Global Fund to Fight AIDS, Tuberculosis and Malaria*. There has been particular support for malaria control (community health workers (CHWs) and long-lasting insecticide-treated net (LLIN) distribution) [4, 5]) for hard-to-reach communities. As a consequence, the national malaria incidence is estimated to have decreased by 49% since 2012: from 8.1 per 1000 to 4.2 per 1000 in 2015 [6].

Community-based malaria management using rapid diagnostic tests (RDTs) and good quality treatment, implemented by CHWs, can substantially improve malaria management in remote communities. CHWs can also distribute LLIN [7]. This approach has been successful in reducing malaria in many settings [8–11] although it has failed in other contexts where community uptake of services was not maintained [12–14]. Maintaining strong community uptake of CHW services is essential to sustain blood examination rates and good malaria control [12, 13, 15, 16] and to provide longitudinal surveillance data from case detection to estimate the true malaria incidence [17]. However, as malaria transmission and incidence falls, the proportion of febrile cases that are malaria, and receive antimalarial treatment, declines correspondingly. Community uptake of CHW programmes that offer only malaria testing and treatment is therefore likely to reduce. Patients attend a health worker because they have fever for which they want treatment. As the probability that a febrile patient has malaria and receives treatment declines, the perceived value of the malaria-only CHW programme declines too, and so the CHWs may become victims of their own success. In order to be appreciated and therefore used by their communities, CHW programmes need to provide relevant services of perceived value and benefit. To achieve this, ‘malaria-only’ CHWs can broaden their service to provide an integrated health care package to address common health problems.

Medical Action Myanmar (MAM), a medical aid organisation, has been supporting a network of CHWs in the most remote communities in Myanmar. These initially provided malaria control activities exclusively. However, as malaria decreased rapidly in these communities, CHWs were trained to offer an extended basic health care (BHC) package in addition to the malaria services. This was initiated in 2013 and 2014 in an effort to meet health needs and to ensure continued community participation with the CHW malaria programme. This extended BHC package incorporated treatment of malnutrition, diarrhoea, and respiratory tract infections, in line with the integrated community case management statement of the World Health Organization (WHO) and United Nations International Children’s Fund (UNICEF) [18]. It also supported referral of severely ill patients and patients suspected to have tuberculosis to the nearest hospital.

All countries in the Greater Mekong Subregion have set national targets for malaria elimination in the near future. Despite interest in integrated CHW programmes, and their proven efficacy in reducing morbidity and mortality from acute respiratory infections [19–23] and diarrhoeal disease [19, 21, 23], there is relatively little information on their efficacy in malaria control and their effect on community uptake in the context of malaria elimination [24]. We conducted a retrospective analysis of 1335 MAM-supported CHWs operating in Myanmar between 2011 and 2016. We assessed the rates of decline in *Plasmodium falciparum* and *Plasmodium vivax* malaria incidence and RDT positivity with each year of CHW operation. In addition, we investigated the effects of the addition of a BHC package on the uptake of malaria services in four cohorts of CHWs which had provided malaria services only for at least 1 year.

## Methods

### Study area and population

**Fig. 1:**
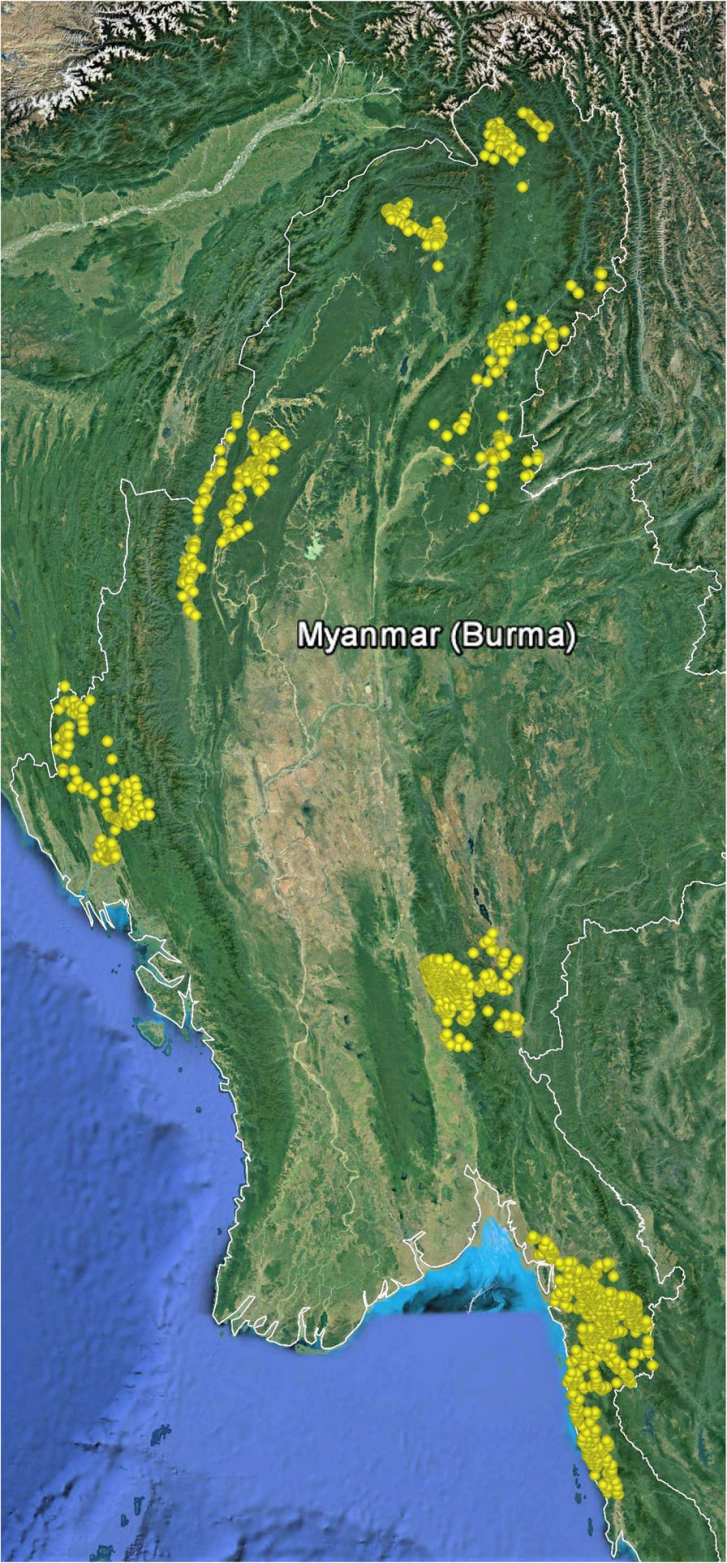
Locations of community health workers within Myanmar supported by Medical Action Myanmar in 2016. Google Earth, US Dept of State Geographer ©2018 Google, Image Landsat/Copernicus, Data SIO, NOAA, US Navy, NGA, GEBCO

Routine monitoring data were obtained from the network of CHWs in remote border areas of Myanmar, adjacent to Thailand, China, India and Bangladesh (Fig. 1). The terrain varies from forested hills to flat areas with many streams and rivers. Yearly rainfall is high (2500–5000 mm/year). During the rainy (malaria) season, many villages are inaccessible. Malaria transmission is heterogeneous ranging from low to high even over small geographic distances and occurs for most of the year with a peak of intensity during and after the rainy season.

In 2011, in cooperation with the Myanmar National Malaria Control Programme, MAM instituted a network of CHWs in remote communities in Mon State, South-East Myanmar, to provide community-based malaria management. Initially, 103 village volunteers were selected after consultation with village leaders and screening for literacy and education level. The volunteers were trained to perform malaria RDTs for all patients in their villages with complaints of fever and to provide treatment accordingly. *P*. *falciparum* malaria was treated with oral artemether-lumefantrine according to national guidelines. This was provided according to body weight in colour-coded blister packages. A single dose of primaquine (0.75mg/kg) was added on the first day of treatment to reduce transmissibility of the infection. *P*. *vivax* malaria was treated with 3 days of chloroquine (25 mg base/kg) plus primaquine, which was given initially once a day (0.25 mg base/kg) for 14 days without G6PD testing. After November 2015, this policy was changed to 15 mg, 30 mg, and 45 mg of primaquine for 5–9 year olds, 10–14 year olds, and 15 year olds respectively once weekly for 8 weeks to mitigate the risk of haemolysis in people who were G6PD deficient. The CHW recorded the test results and treatment provided, together with basic patient information details on standard forms, and these reports were provided to the regional Vector Borne Disease Control office each month. CHWs agreed to be available in their village every day for a minimum of 1 h to provide diagnosis and treatment, usually early morning or late afternoon when villagers returned from their fields. The CHWs received a fixed monthly incentive (≈$4 USD) and a small incentive (≈$0.4 USD) for every patient tested (Additional file 1). All activities were supported by health education and community engagement discussions to inform the community and to seek their feedback.

During 2013 and 2014, it became clear that the incidence of malaria had decreased markedly since the introduction of malaria-focused CHWs and that other important unaddressed health needs had become relatively more common and pressing. A BHC package was therefore added to the CHW malaria services which comprised four components:

1. Management of selected common diseases, including respiratory tract infections, diarrhoea, and skin infections
2. Detection of acute malnutrition with mid-upper-arm-circumference measurement and treatment with ready-to-use therapeutic food (plumpy’nut™)
3. Active case finding of patients suspected to have tuberculosis and referral to the nearest government hospital
4. Referral of complicated and severely ill patients with life threatening or disabling diseases to the nearest government hospital

Over the next 2 years, the network of CHWs trained expanded and, by the end of 2016, had grown to 1326. The CHWs received training in line with the curricula of the National Malaria Control Programme and National TB Programme (further training details in Additional file 1).

### Statistical analysis

To assess temporal changes in incidence, RDT positivity rates and monthly blood examination rates, negative binomial mixed effects regression models were constructed with random intercepts and slopes. Seasonal variations in outcomes were accounted for by the inclusion of harmonic functions of time as a covariate. As a sensitivity analysis, we fitted alternate models with (1) adjustment for seasonality with a categorical month indicator, (2) no adjustment for seasonality, and (3) adjustment with a first-order autoregressive variance structure (Additional file 2). Plots of residuals were inspected for signs of autocorrelation. When modelling incidence and RDT positivity rates, the outcome was RDT-positive results (per month, per CHW) and the primary exposure was years of CHW operation. The exposure variable for the incidence model was the population served by a CHW, and the exposure variable for the RDT positivity rate model was the number of RDTs performed.

When modelling monthly blood examination rates, the population each CHW served was incorporated as the exposure variable and the outcome variable was total RDTs performed. To assess the impact of the introduction of BHC package services on CHW blood examination rate, we adopted standard methods for interrupted time series analysis [25]. We identified cohorts of CHWs from the same state/region where at least ten CHWs had been providing malaria-only services for more than 12 months and where these CHWs received BHC package training over 2 months. The announcement and introduction of BHC services was expected to affect consultation rates immediately, so we made the a priori decision to construct a slope and level change model, estimating the immediate change and change in time trend at time of BHC implementation. A likelihood ratio test comparing a level change model (BHC main effect only) to the slope and level change model provided a *p* value for the change in slope at BHC implementation. Statistical analyses were performed using Stata 14.2 (StataCorp, College Station, TX) and R software (version 3.4.4, The R Foundation for Statistical Computing, Vienna, Austria).

## Results

### CHW recruitment and population coverage

In 2011, a group of 103 CHWs were recruited, trained, and supplied to diagnose and treat malaria in remote communities in Mon State, Myanmar, covering a population of 163,171. This network of CHWs was expanded subsequently, and over the next 5 years, 1335 CHWs were trained and supplied. Of these, 1326 (98%) were still operational in 2016 in the following states and divisions: Mon, Kayah, Kayin, and Thanintharyi in the East; Kachin and Sagaing in the North; and Chin and Rakhine in the West, covering an estimated population of 728,057 (Fig. 1). This is approximately 1.3% of the Myanmar population.

Over this period, 571,286 RDTs were performed by the CHWs and 10,604 *P*. *falciparum* infections and 10,657 *P*. *vivax* infections were detected and treated. The median (interquartile range) overall blood examination rate (i.e. percentage of the total population who had blood tested) across all CHWs was 4.2% (1.9–7.6%) per month. The populations serviced by individual CHWs were generally small (median 309 (IQR 170–597)). Median monthly blood examination rates were higher in CHWs serving smaller populations: 7.5% (IQR 4.3–12.6%) for CHWs with a catchment population less than 250, and 4.2% (IQR 2.4–6.3%) for those with a catchment population of 250–500 compared with 1.6% (IQR 0.8%–2.9%) for those with a catchment population greater than 500.

### *P*. *falciparum* and *P*. *vivax* incidence and RDT positivity rates

**Fig. 2:**
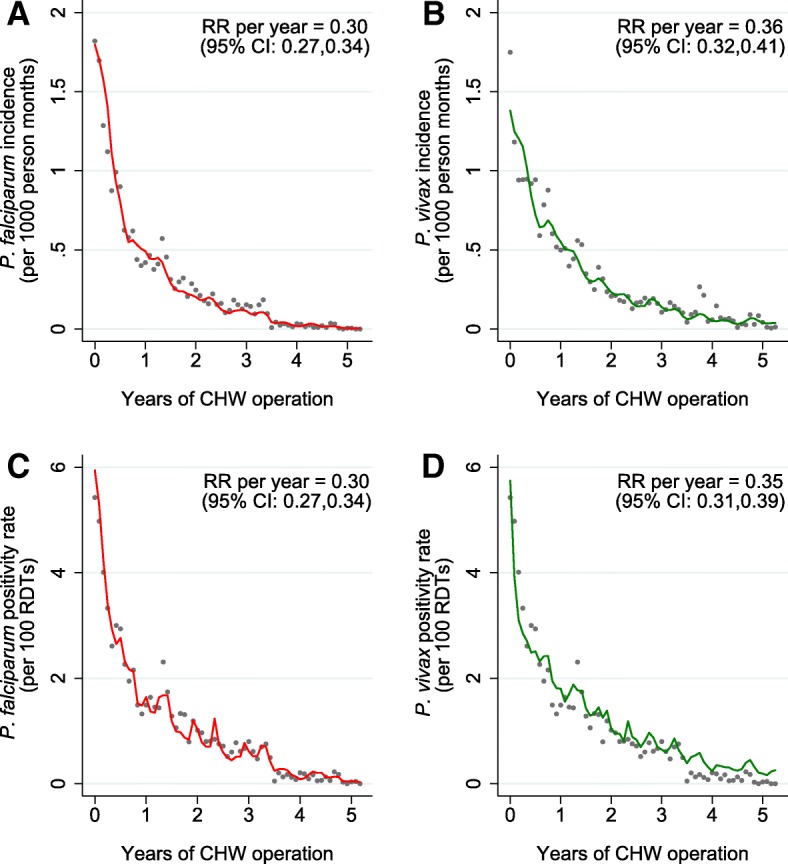
Malaria incidence and RDT positivity rates by years of CHW operation. Grey dots denote observed aggregated data, and the line indicates the prediction from a mixed effects negative binomial regression model for **a** *P. falciparum* incidence (per 1000 person months), **b** *P*. *vivax* incidence (per 1000 person months), **c** *P*. *falciparum* RDT positivity rate (%), and **d** *P. vivax* RDT positivity rate (%). Models were constructed from activities of 1335 CHWs and 571,286 RDT results. CHW: community health worker, RR: rate ratio, 95% CI: 95% confidence interval as calculated from the model

There was considerable heterogeneity in the initial incidence and positivity rates of *P*. *falciparum* and *P*. *vivax* in newly opened CHW posts (Table 1, Additional files 3 and 4). Across the study villages, the incidence and RDT positivity rates of both *P*. *falciparum* and *P*. *vivax* malaria declined markedly after institution of the CHWs (Fig. 2). For each year of CHW operation, there was a 70% (95% CI 66–73%) decrease in *P*. *falciparum* incidence (Fig. 2a) and a 64% (95% CI 59–68%) decrease in *P*. *vivax* incidence (Fig. 2b). Similar declines in RDT positivity rates were observed; each year of CHW operation was associated with a 70% (95% CI 66–73%) decrease in the *P*. *falciparum* (Fig. 2c) and a 65% (95% CI 61–69%) decrease in the *P*. *vivax* RDT positivity rates (Fig. 2d). Similar declines in incidence and RDT positivity rates were obtained from three sensitivity analyses, which (1) accounted for seasonality with a monthly indicator, (2) did not adjust for seasonality, and (3) accounted for seasonality with a first-order autoregressive variance structure (Additional file 2). In sensitivity analysis 3, estimates of the decline in *P*. *falciparum* incidence, *P*. *vivax* incidence, and *P*. *vivax* RDT positivity rates were of slightly smaller magnitude (64%, 60%, and 62% declines respectively) than estimates obtained in the primary analysis.

**Table 1:** Malaria in the first year of community health worker operation in eight regions of Myanmar

Region	Programme started	*P*. *falciparum*		*P*. *vivax*	
		Positivity rate^a^	Incidence^b^	Positivity rate^a^	Incidence^b^
Chin	May 2016^c^	22.6	14.7	4.3	2.8
Kachin	April 2014	0.6	0.1	0.8	0.2
Kayah	August 2014	1.3	0.3	3.9	0.8
Kayin	March 2012	11.7	5.3	16.9	7.7
Mon	September 2011	2.8	0.4	5.1	0.7
Rakhine	November 2016^c^	1.0	0.1	0	0
Sagaing	March 2016^c^	1.3	0.2	0.4	0.1
Tanintharyi	March 2014	0.1	0.04	0.2	0.1

^a^Per 100 rapid diagnostic tests. ^b^Per 1000 person months. ^c^Summary data covers months from start date until December 2016

### Blood examination rates before and after the introduction of a basic health care package

Training of CHWs to provide the BHC package in addition to the malaria activities began in April 2013 with 43 CHWs. By the end of 2016, 1040 CHWs were providing a BHC package. In 2016, CHWs provided 284,658 BHC consultations, including diagnosis of 14,509 cases of pneumonia and identification and referral to the nearest hospital of 6278 patients with suspected tuberculosis and 859 patients for other severe disease causes.

The monthly blood examination rates of the four cohorts of CHWs that introduced the BHC package after providing malaria-only services for at least a year were investigated. These cohorts comprised 154 CHWs who were all based in Eastern Myanmar (three cohorts in Mon state and one cohort in Kayin state, Table 2). The RDT positivity rate before BHC package introduction was highest in the Kayin cohort (18.1% and 11.4% respectively, in the 2 years preceding BHC), while the cohorts from Mon State had lower RDT positivity rates (7.4% and 6.2%; 9.4% and 5.4%; and 1.8% and 0.9% in the 2 years preceding the introduction of the BHC package).

**Table 2:** Characteristics of the cohorts included in interrupted time series analysis

	Cohort 1	Cohort 2	Cohort 3	Cohort 4
Number of CHWs	44	18	19	73
Total monthly reports	2759	1039	1214	3252
Total RDTs performed	60,461	13,904	33,585	64,909
State	Mon	Mon	Mon	Kayin
Townships	Kyaikmaraw, Ye	Ye	Kyaikmaraw, Mudon, Paung	Kyainseikgyi
Date first CHW operational	September 2011	September 2011	September 2011	March 2012
Dates BHC package introduced	May–June 2013	August–September 2013	March 2014	May 2014
Total population covered	47,831	6415	31,724	54,595
Median (IQR) community population	850 (387–1459)	388 (259–430)	1285 (905–2644)	548 (281–870)
MBER (year prior to BHC)	1.2%	1.7%	1.5%	3.4%
RDT (+) rate (1–2 years prior to BHC)	7.4%	9.4%	1.8%	18.1%
RDT (+) rate (year prior to BHC)	6.2%	5.4%	0.9%	11.4%
RDT (+) rate (year post-BHC)	1.4%	0.8%	0.3%	6.0%

*CHW* community health worker, *RDT* rapid diagnostic test for malaria, *BHC* basic health care, *MBER* monthly blood examination rate, *IQR* interquartile range

**Fig. 3:**
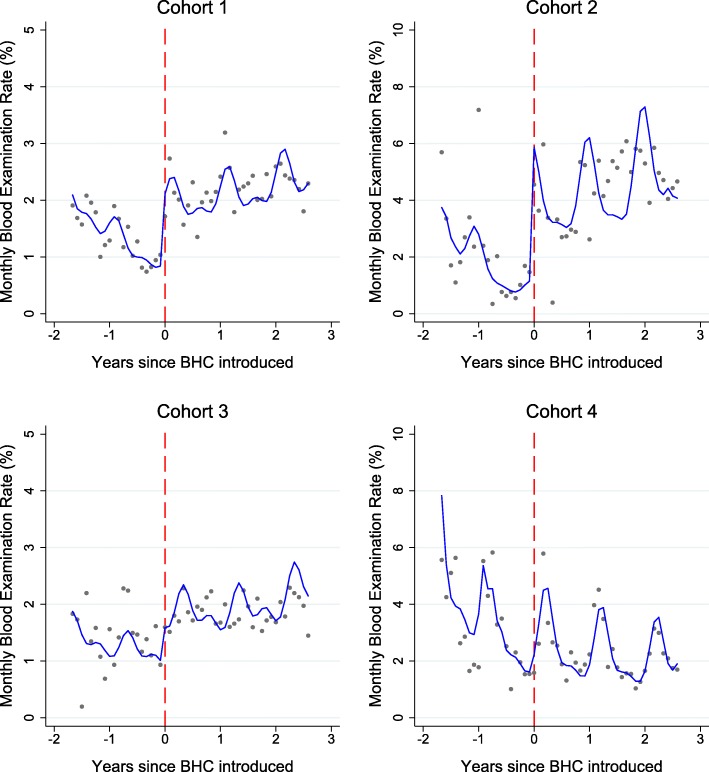
Monthly blood examination rates in four cohorts of CHWs pre/post addition of basic health care services. Grey dots represent observed aggregated data, and the blue line indicates the prediction from a mixed effects negative binomial regression model. Vertical red line denotes the time when the basic health care package was introduced. Graphs are displayed for time points where data were available from all cohorts for consistency. BHC: basic health care. For model coefficients, see Table 3

In the pre-introduction period when the CHWs provided malaria services only, cohorts 1, 2, and 4 were experiencing significant declines in blood examination rates (*p* < 0.0001 for all) while in cohort 3 the decline was small and was not significant (*p* = 0.91; Fig. 3, Table 3). The introduction of the BHC package was associated with an immediate step-change increase in the blood examination rate in all four cohorts, (rate ratios post-BHC relative to pre-BHC: 2.3, 5.4, 1.7, and 1.1 respectively, *p* < 0.0001 for cohorts 1 to 3 and *p* = 0.16 for cohort 4, Fig. 3, Table 3). The three cohorts (1, 2, and 4) that experienced significant blood examination rate declines prior to BHC implementation experienced significant positive changes in slope after BHC implementation (likelihood ratio test for interaction *p* < 0.0001, *p* < 0.0001, and *p* = 0.01 respectively), while in cohort 3 blood examination rates were stable over time prior to BHC implementation and did not deteriorate with time after the step-change increase. We repeated the interrupted time series analysis of blood examination rates in three sensitivity analyses, which (1) accounted for seasonality with a monthly indicator, (2) did not adjust for seasonality, and (3) accounted for seasonality with a first-order autoregressive variance structure (Additional file 2). Results were broadly similar across these sensitivity analyses with the exception of the estimate of the step change in cohort 4 (rate ratio = 1.1 in the primary analysis and in sensitivity analysis 1; rate ratio = 1.5 in sensitivity analysis 2 and sensitivity analysis 3). Across the entire programme, CHWs offering a malaria-only service had an average blood examination rate of 1.63 per 100 persons per month (158,425 RDTs over 9.72 × 10^6^ person months). CHWs offering a BHC package had an average blood examination rate of 3.20 per 100 persons per month (412,861 RDTs over 1.29 × 10^7^ person months).

**Table 3:** Interrupted time series analysis: monthly blood examination rates pre/post-basic health care package introduction

Cohort	Pre-BHC trend (per year) Rate Ratio (95% CI); *p* value	Step change at BHC introduction Rate ratio (95% CI); *p* value	Post-BHC trend (per year) Rate ratio (95% CI); *p* value	*p* value for change in trend^a^
1	0.60 (0.52,0.69); < 0.0001	2.28 (1.97,2.64); < 0.0001	1.08 (1.01,1.16); 0.03	< 0.0001
2	0.40 (0.31,0.53); < 0.0001	5.38 (3.96,7.32); < 0.0001	1.01 (0.87,1.18); 0.86	< 0.0001
3	0.99 (0.83,1.17); 0.91	1.71 (1.41,2.08); < 0.0001	1.03 (0.88,1.22); 0.71	0.51
4	0.76 (0.66,0.86); < 0.0001	1.10 (0.96,1.26); 0.16	0.89 (0.82,0.96); 0.002	0.01

*BHC* basic health care, *CI* confidence interval

^a^Change in trend from pre-BHC to post-BHC

## Discussion

Malaria elimination is now firmly on the agenda in much of the tropical world [26], and many countries, particularly in Southeast Asia, have set ambitious timelines for achieving it. In Myanmar, as in many low- and middle-income countries, CHWs are key to the delivery of malaria diagnosis and treatment in remote areas. This retrospective analysis clearly demonstrates the substantial public health benefit, in terms of reduced malaria incidence, of instituting trained CHWs in remote malaria-affected villages. The two-thirds annual reduction in incidence in these communities compares with an estimated decrease of 20% per year for the overall Myanmar yearly malaria incidence between 2012 and 2015 [6]. Thus, with relatively little training, but reliable supplies and careful monitoring, these community members provided a popular and highly effective public health service. The most likely explanation for the CHWs’ success in reducing malaria is that provision of community-based early diagnosis and effective quality-assured treatment, coupled with LLIN distribution, significantly reduced malaria transmission.

The continued function of CHWs is essential to drive malaria to elimination, and to ensure continued monitoring of any imported malaria cases once local transmission has ceased. But having achieved large reductions in malaria, the CHW who treats only malaria becomes increasingly inactive and irrelevant as fewer and fewer patients with acute febrile illness are diagnosed with malaria and receive specific treatment. From the febrile patient’s perspective, it matters little what the cause of their fever is, as long as it is treated effectively, so the incentive to seek treatment from the “malaria only” CHW declines as malaria incidence declines. Malaria-only community health workers recognise that when malaria declines, febrile patients are less likely to consult them, and wish to be able to provide help to patients with non-malaria fever [27]. Patients return to untrained informal health care providers, who provide inappropriate medicines and interventions. This was our concern when the numbers of patients seeking malaria RDTs in remote Myanmar villages declined.

To improve access to health care and to remedy the declining consultation rate, the remit of the CHWs was broadened to include other common febrile illnesses and referral of severely ill patients to the nearest hospital. This initiative was welcomed by the communities as non-malaria febrile illnesses now received a specific treatment as well, and for malaria control activities, it had the major advantage of sustaining quality. In this retrospective assessment, there was a decrease in health seeking behaviour and malaria RDT testing after malaria transmission had gone down, but this trend reversed after broadening the health care package of the CHWs. A limitation of this study was that it was observational, not experimental. However, the consistency and magnitude of the estimates observed, across four separate cohorts, suggest that the improvements in malaria RDT testing rates are real, accurate, and representative of what could be expected in future implementations of integrated care. Of note, we observed different magnitudes of effect across different cohorts, suggesting that the context of a given community will be an important factor in service uptake. Cohort 4 had the smallest magnitude step-increase in blood examination rate, though in sensitivity analyses this estimate was higher when seasonality was not accounted for, or was accounted for using first-order autoregression (Additional file 2). Cohort 4 also had the highest malaria RDT positivity rate prior to BHC package introduction; a small step-increase was consistent with an expectation that this intervention will be most effective in communities that feel malaria is no longer a primary concern. Interrupted time series analyses are well suited to the evaluation of intervention effects in real-world settings [28], and as it seems unlikely that the incidence of febrile illnesses changed coincidentally with the change in CHW practice, a causal relationship is likely.

Most health professionals recognise that CHWs can have an important positive effect on malaria diagnosis and treatment in the community and that the success of community case management can be replicated for other common diseases. WHO and UNICEF outlined their support for integrated community case management in 2012 [18], noting that appropriately trained, supervised, and supported CHWs can identify and correctly treat most childhood respiratory tract infections and diarrhoea [29]. It has been estimated that community management of childhood pneumonia could reduce mortality from pneumonia in children less than 5 years old by 70% [22]. Oral rehydration salts and zinc reduce the mortality of diarrhoeal disease in community settings; community promotion of oral rehydration salts was estimated to reduce the number of deaths due to diarrhoea by 69% (95% CI 51–80%) [30], and zinc supplementation is estimated to decrease diarrhoea mortality by 23% (95% CI 15–31%) [31]. The approach of integrating disease-specific programmes with other health services (the “diagonal approach”) is likely to be appropriate for other narrow disease-specific programmes, which can also see their sustainability threatened by their own success, and is critical for effective health systems strengthening [32, 33].

During the period of this study, financial incentive schemes for CHWs in Myanmar varied across different organisations; the value of incentives offered by MAM was neither the highest nor the lowest, among organisations supporting Myanmar CHWs [34]. There are concerns that per test incentives that are too high can have a negative influence on CHW diagnostic practices. Conversely, when incentives are too low or absent, the CHW may stop testing altogether and retention may become difficult. More research is needed to identify the correct balance of monthly incentive and test incentive in low-income areas. Ultimately, frequent monitoring and supervision are essential under any incentive scheme to maintain high standards of care and to check for aberrant practices.

Some policy makers worry that CHWs are not capable of providing the correct diagnosis for patients with non-malaria fevers. In several countries, CHWs are allowed to prescribe antimalarials but not antibiotics. Health professionals fear that CHWs may become the new generation of informal health care providers, overprescribing unnecessary antibiotics without proper diagnosis. Instead, they suggest that it is better to refer patients with non-malaria febrile illnesses to the nearest government health service. However, in remote areas with poor infrastructure, numerous barriers of geographical access, availability, affordability, and acceptability hamper access to government health services [35, 36], and such a strategy is simply not feasible. Even when trusted CHWs refer patients to the nearest hospital, many will not go [37, 38]. Smaller villages are generally more remote and face more barriers to health service access so typically have a higher uptake of CHW services. It is in these villages that integration of community-based health services is most important. Integrated CHWs can be carefully regulated, which could diminish irrational antibiotic use, prevent antimicrobial resistance, and substantially reduce morbidity and mortality in a cost-effective manner.

This retrospective analysis includes over half a million malaria RDT results from a large number of communities (1335) over 5 years. The downward trends of incidence for both *P*. *falciparum* and *P*. *vivax* malaria after the introduction of community-based malaria management are substantial and convincing. By contrast, the analysis of the impact of the introduction of a BHC package was performed only in a relatively small number of cohorts (four) including 154 communities. This was unavoidable as the number of CHWs with enough data before and after introduction of the BHC package was limited, but the results are likely to be relevant to other remote communities.

## Conclusions

In summary, this study demonstrates the clear benefits of the integration of basic health care into CHW services to sustain uptake of malaria services. Malaria has decreased significantly over the past years, and a further decline of morbidity and mortality is expected. As malaria approaches elimination, National Malaria Control Programmes must plan for likely reductions in funding and ensure that the capabilities for vector control and malaria diagnosis and treatment are maintained. A diagonal approach to health systems strengthening is needed to ensure effective, sustainable, and cost-effective health services in remote communities. A CHW for malaria-only is not sustainable and must become the CHW for an integrated package of common health problems. This will benefit the community and will ensure that malaria is eliminated and then stays eliminated.

## Additional files

Additional file 1: Community Health Workers Training, Monitoring and Incentives: Additional details of the training, monitoring and incentive structure of Medical Action Myanmar community health workers. (DOCX 13 kb)

Additional file 2: Sensitivity analyses. Sensitivity analyses of models of malaria incidence and RDT positivity rates by years of CHW operation and of the interrupted times series analysis of basic health care package introduction. (DOCX 20 kb)

Additional file 3: Malaria incidence and RDT positivity rates by calendar year for Kachin, Kayah, Mon, Rakhine, Sagaing, Tanintharyi. (A) *P. falciparum* incidence (per 1000 person months); (B) *P. vivax* incidence (per 1000 person months); (C) *P. falciparum* RDT positivity rate (%); (D) *P. vivax* RDT positivity rate (%). RDTs = Rapid Diagnostic Tests. Chin and Kayin are presented separately in Additional file 3 for legibility. (PDF 160 kb)

Additional file 4: Malaria incidence and RDT positivity rates by calendar year for Chin and Kayin. (A) *P. falciparum* incidence (per 1000 person months); (B) *P. vivax* incidence (per 1000 person months); (C) *P. falciparum* RDT positivity rate (%); (D) *P. vivax* RDT positivity rate (%). RDTs = Rapid Diagnostic Tests. Other state/regions are presented separately in Additional file 2 for legibility. (PDF 146 kb)

## Abbreviations

BHC: Basic health care
CHW: Community health worker
CI: Confidence interval
IQR: Interquartile range
MAM: Medical Action Myanmar
RDT: Rapid diagnostic test
UNICEF: United Nations International Children’s Fund
WHO: World Health Organization
