# Supplementary material for: Malaria elimination in remote communities requires integration of malaria control activities into general health care: an observational study and interrupted time series analysis in Myanmar
Source: BMC Med. 2018 Oct 22;16:183. doi: 10.1186/s12916-018-1172-x (PMC6196466; doi:10.1186/s12916-018-1172-x)
Supplement: Supplementary file 1 — Community Health Workers Training, Monitoring and Incentives: Additional details of the training, monitoring and incentive structure of Medical Action Myanmar community health workers. (DOCX 13 kb) [file 12916_2018_1172_MOESM1_ESM.docx]

**Community Health Worker’s Training, Monitoring and Incentives**

Initially the CHWs received two days class room teaching in malaria management, following the National Malaria Control Programme curriculum. This included recognition of symptoms of uncomplicated and complicated malaria, RDT testing practice, the treatment protocol, reporting and referral criteria. An exam was administered at the conclusion of the training, CHWs were then supplied with RDTs and medicines. Classroom teaching was followed by monthly on-the-job training sessions in the remote villages, together with MAM professional health staff.

Tuberculosis active case finding training took one day, following the curriculum of the National TB programme. During monthly on-the-job training all patients with suspected TB were discussed and referred when appropriate. Recognition and management of specific common diseases, malnutrition and referral of complicated and severely ill patients was also taught for two days in the class room followed by intense on-the-job training by a MAM doctor, which was done during monthly mobile clinics held in the remote communities. During these mobile clinics all patients with complaints were invited to attend, and their complaints were investigated and discussed between the MAM doctor and the CHW. Patients with chronic complaints who had visited the CHW during the month, were invited to consult the doctor during these monthly visits.

Acute and severely ill patients were eligible for immediate referral. Ideally, these patients would be discussed first with the MAM doctor by phone, in order to determine if referral was appropriate, but this was only possible with CHWs who had access to a phone network. If a MAM doctor could not be contacted, the CHWs could decide by themselves if a referral was required, with the help of referral guidelines. In principle, all patients with a life threatening treatable disease or with a treatable disease that could cause permanent disability were entitled to referral. All costs involved for transport, food and treatment were provided. MAM ‘referral-staff’ followed patients in the hospitals, supported their needs and facilitated their return trip home. The monthly mobile clinic visits were also used to monitor the quality of the activities of the CHW during the previous month. Some patients were selected randomly and visited at home and the quality of the diagnosis and management of these patients were reviewed. Reports were analyzed and discussed, the use of diagnostics and medicines were reviewed, their stocks were inspected, and these were re-supplied as needed.

Throughout the course of the programme, CHWs received a fixed monthly incentive (5000MMK) and a smaller incentive for each RDT administered (initially 300MMK and then 500MMK from January 2015, capped at 90 tests per month). In USD terms, the monthly incentive was approximately 6USD at the beginning of the programme and 3.7USD at the conclusion of 2016 and the RDT incentive cost was approximately $US0.37 at the beginning of the programme and $US0.37 at the conclusion of 2016.
