# Supplementary material for: Malaria elimination in remote communities requires integration of malaria control activities into general health care: an observational study and interrupted time series analysis in Myanmar
Source: BMC Med. 2018 Oct 22;16:183. doi: 10.1186/s12916-018-1172-x (PMC6196466; doi:10.1186/s12916-018-1172-x)
Supplement: Supplementary file 2 — Sensitivity analyses. Sensitivity analyses of models of malaria incidence and RDT positivity rates by years of CHW operation and of the interrupted times series analysis of basic health care package introduction. (DOCX 20 kb) [file 12916_2018_1172_MOESM2_ESM.docx]

**Sensitivity Analyses**

Our models reported in the manuscript included harmonic functions of time as a covariate to account for seasonality. As a sensitivity analysis we fit alternate models with:

(1) Adjustment for seasonality with a categorical month indicator as a covariate.

(2) No adjustment for seasonality.

(3) Seasonality accounted for with a first-order autoregressive variance structure.

The original model, sensitivity analysis model 1 and sensitivity analysis model 2 were fit in Stata. Sensitivity analysis model 3 was fit using the glmmTMB package in R.

For sensitivity analyses of the analysis of malaria incidence and RDT positivity rates by years of CHW operation (Table 1), estimates and confidence intervals were similar when adjusting for seasonality with a categorical month indicator (sensitivity analysis model 1) and when ignoring seasonality (sensitivity analysis model 2). The magnitude of the main effect was of slightly smaller magnitude in model 3 for three of the four outcomes.

For sensitivity analyses of the interrupted time series analysis (Table 2), estimates and confidence intervals for the four cohorts were similar when adjusting for seasonality with a categorical month indicator (sensitivity analysis model 1). When ignoring seasonality (sensitivity analysis model 2) or modelling seasonality with first order autoregression (sensitivity analysis model 3), the magnitude of the estimate of the step-change in cohort 4 increased.

**Table 1. Sensitivity analyses of models of malaria incidence and RDT positivity rates by years of CHW operation.**

| **Outcome** | **Rate ratio (95% Confidence Interval) per year of CHW operation** | | | |
| --- | --- | --- | --- | --- |
|  | **Original model** | **Sensitivity analysis model 1** | **Sensitivity analysis model 2** | **Sensitivity analysis model 3** |
| ***P. falciparum* incidence** | 0.30 [0.27,0.34] | 0.30 (0.27,0.34) | 0.31 (0.27,0.34) | 0.36 (0.30,0.41) |
| ***P. vivax* incidence** | 0.36 [0.32,0.41] | 0.36 (0.32,0.40) | 0.36 (0.32,0.41) | 0.40 (0.35,0.46) |
| ***P. falciparum* RDT positivity rate** | 0.30 [0.27,0.34] | 0.30 (0.27,0.34) | 0.31 (0.27,0.34) | 0.30 (0.24,0.36) |
| ***P. vivax* RDT positivity rate** | 0.35 [0.31,0.39] | 0.35 (0.31,0.39) | 0.35 (0.31,0.40) | 0.38 (0.32,0.44) |

**Table 2. Sensitivity analyses of interrupted time series analysis: monthly blood examination rates pre/post basic health care package introduction.**

| **Cohort** | **Pre-BHC trend (per year) Rate Ratio (95% CI); p value** | **Step change at BHC introduction**  **Rate Ratio (95% CI); p value** | **Post-BHC trend (per year)**  **Rate Ratio (95% CI); p value** | **p value for change in trend^a^** |
| --- | --- | --- | --- | --- |
| **Original model** | | | | |
| 1 | 0.60 (0.52,0.69); <0.0001 | 2.28 (1.97,2.64); <0.0001 | 1.08 (1.01,1.16); 0.03 | <0.0001 |
| 2 | 0.40 (0.31,0.53); <0.0001 | 5.38 (3.96,7.32); <0.0001 | 1.01 (0.87,1.18); 0.86 | <0.0001 |
| 3 | 0.99 (0.83,1.17); 0.91 | 1.71 (1.41,2.08); <0.0001 | 1.03 (0.88,1.22); 0.71 | 0.51 |
| 4 | 0.76 (0.66,0.86); <0.0001 | 1.10 (0.96,1.26); 0.16 | 0.89 (0.82,0.96); 0.002 | 0.01 |
| **Sensitivity analysis model 1** | | | | |
| 1 | 0.60 (0.52,0.69); <0.0001 | 2.26 (1.95,2.62); <0.0001 | 1.08 (1.01,1.17); 0.03 | <0.0001 |
| 2 | 0.41 (0.31,0.54); <0.0001 | 5.26 (3.87,7.15); <0.0001 | 1.02 (0.88,1.19); 0.79 | <0.0001 |
| 3 | 0.99 (0.83,1.17); 0.90 | 1.71 (1.41,2.08); <0.0001 | 1.03 (0.88,1.22); 0.69 | 0.49 |
| 4 | 0.75 (0.66,0.85); <0.0001 | 1.10 (0.97,1.26); 0.14 | 0.90 (0.83,0.97); 0.005 | 0.004 |
| **Sensitivity analysis model 2** | | | | |
| 1 | 0.59 (0.51,0.68); <0.0001 | 2.46 (2.12,2.85); <0.0001 | 1.07 (1.00,1.15); 0.06 | <0.0001 |
| 2 | 0.42 (0.32,0.55); <0.0001 | 4.84 (3.60,6.52); <0.0001 | 1.01 (0.87,1.17); 0.90 | <0.0001 |
| 3 | 1.02 (0.86,1.21); 0.84 | 1.63 (1.34,1.98); <0.0001 | 1.04 (0.89,1.23); 0.60 | 0.69 |
| 4 | 0.64 (0.56,0.74); <0.0001 | 1.45 (1.26,1.67); <0.0001 | 0.85 (0.79,0.91); <0.0001 | <0.0001 |
| **Sensitivity analysis model 3** | | | | |
| 1 | 0.57 (0.47,0.69); <0.0001 | 2.65 (2.18,3.23); <0.0001 | 1.07 (0.98,1.17); 0.11 | <0.0001 |
| 2 | 0.41 (0.29,0.58); <0.0001 | 5.51 (3.70,8.21); <0.0001 | 1.09 (0.92,1.29); 0.31 | <0.0001 |
| 3 | 0.89 (0.70,1.14); 0.35 | 1.57 (1.17,2.12); 0.003 | 1.08 (0.87,1.35); 0.49 | 0.30 |
| 4 | 0.63 (0.53,0.74); <0.0001 | 1.48 (1.22,1.80); <0.0001 | 0.86 (0.78,0.95); 0.002 | 0.002 |

BHC = Basic Health Care, CI = Confidence Interval

^a^Change in trend from pre-BHC to post-BHC
