# Supplementary figures and images for: Malaria elimination in remote communities requires integration of malaria control activities into general health care: an observational study and interrupted time series analysis in Myanmar
Source: BMC Med. 2018 Oct 22;16:183. doi: 10.1186/s12916-018-1172-x (PMC6196466; doi:10.1186/s12916-018-1172-x)

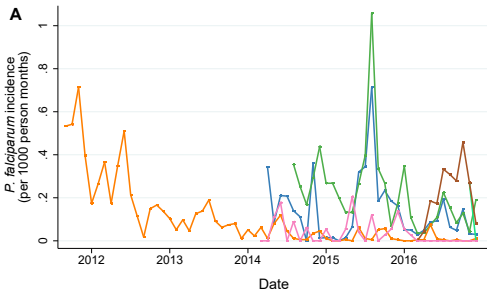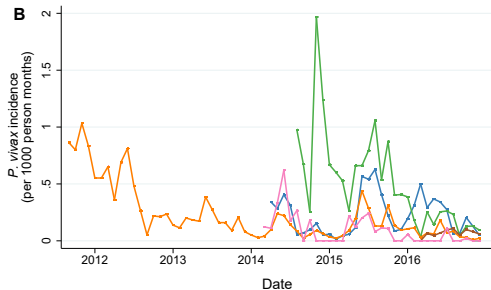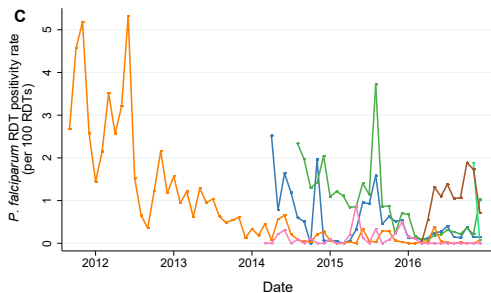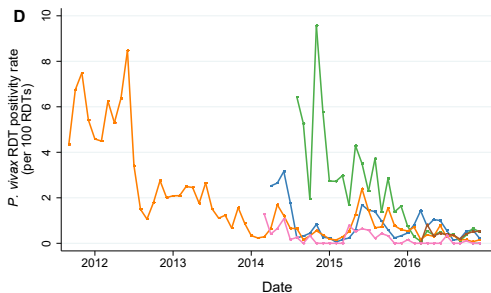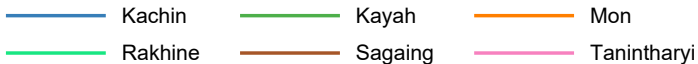

Supplement: Supplementary file 3 — Malaria incidence and RDT positivity rates by calendar year for Kachin, Kayah, Mon, Rakhine, Sagaing, Tanintharyi. (A) P. falciparum incidence (per 1000 person months); (B) P. vivax incidence (per 1000 person months); (C) P. falciparum RDT positivity rate (%); (D) P. vivax RDT positivity rate (%). RDTs = Rapid Diagnostic Tests. Chin and Kayin are presented separately in Additional file 3 for legibility. (PDF 160 kb) [file 12916_2018_1172_MOESM3_ESM.pdf]

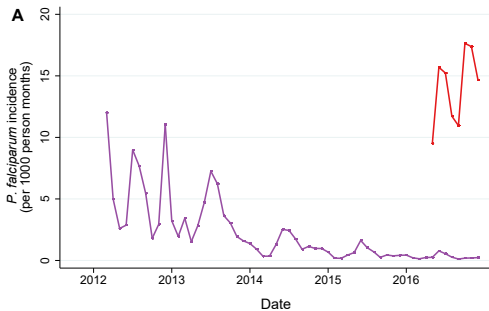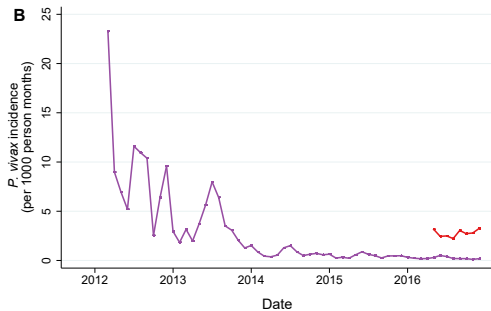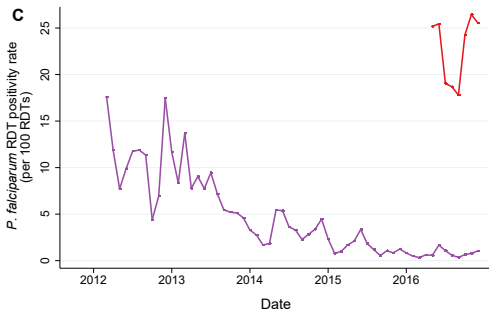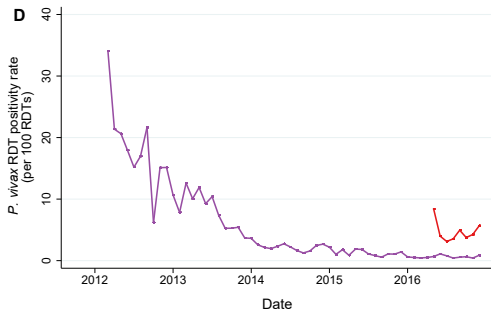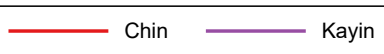

Supplement: Supplementary file 4 — Malaria incidence and RDT positivity rates by calendar year for Chin and Kayin. (A) P. falciparum incidence (per 1000 person months); (B) P. vivax incidence (per 1000 person months); (C) P. falciparum RDT positivity rate (%); (D) P. vivax RDT positivity rate (%). RDTs = Rapid Diagnostic Tests. Other state/regions are presented separately in Additional file 2 for legibility. (PDF 146 kb) [file 12916_2018_1172_MOESM4_ESM.pdf]
